# Matrix metalloproteinase inhibitors enhance the efficacy of frontline drugs against *Mycobacterium tuberculosis*

**DOI:** 10.1371/journal.ppat.1006974

**Published:** 2018-04-26

**Authors:** Yitian Xu, Lihua Wang, Matthew D. Zimmerman, Kai-Yuan Chen, Lu Huang, Dah-Jiun Fu, Firat Kaya, Nikolai Rakhilin, Evgeniya V. Nazarova, Pengcheng Bu, Veronique Dartois, David G. Russell, Xiling Shen

**Affiliations:** 1 Department of Biological and Environmental Engineering, Cornell University, Ithaca, NY, United States of America; 2 Public Health Research Institute, New Jersey Medical School, Rutgers, The State University of New Jersey, Newark, NJ, United States of America; 3 School of Electrical and Computer Engineering, Cornell University, Ithaca, NY, United States of America; 4 Department of Biomedical Engineering, Duke University, Durham, NC, United States of America; 5 Department of Microbiology and Immunology, Cornell University, Ithaca, NY, United States of America; 6 Department of Biomedical Sciences and Cornell Stem Cell Program, Cornell University, Ithaca, NY, United States of America; 7 Key Laboratory of RNA Biology, Key Laboratory of Protein and Peptide Pharmaceutical, Institute of Biophysics, Chinese Academy of Sciences, Beijing, China; National Institutes of Health, UNITED STATES

## Abstract

*Mycobacterium tuberculosis* (Mtb) remains a grave threat to world health with emerging drug resistant strains. One prominent feature of Mtb infection is the extensive reprogramming of host tissue at the site of infection. Here we report that inhibition of matrix metalloproteinase (MMP) activity by a panel of small molecule inhibitors enhances the *in vivo* potency of the frontline TB drugs isoniazid (INH) and rifampicin (RIF). Inhibition of MMP activity leads to an increase in pericyte-covered blood vessel numbers and appears to stabilize the integrity of the infected lung tissue. In treated mice, we observe an increased delivery and/or retention of frontline TB drugs in the infected lungs, resulting in enhanced drug efficacy. These findings indicate that targeting Mtb-induced host tissue remodeling can increase therapeutic efficacy and could enhance the effectiveness of current drug regimens.

## Author summary

*Mycobacterium tuberculosis* (Mtb) continues to be the leading cause of death from a single infectious agent worldwide, leading to 1.8 million deaths in 2015. The long treatment required (6–9 months), with all of its incumbent problems, can promote the emergence of multidrug-resistant (MDR) TB strains, so strategies to shorten the treatment duration are in dire need. Mtb’s success as a pathogen hinges on its ability to remodel the host tissue, characterized by extracellular matrix (ECM) deposition and leaky vascularization. Here we report that inhibition of matrix metalloproteinases (MMPs) significantly enhances the potency of frontline TB antibiotics. These MMP inhibitors increase the relative proportion of healthy blood vessels versus leaky dysfunctional vessels at the infection site, and enhance drug delivery and/or retention. Our study highlights the potential of targeting Mtb-induced host tissue remodeling to enhance the efficacy of current frontline antibiotics. It also suggests an alternative therapeutic strategy to repair the leaky blood vessels in TB granulomas to enhance drug delivery. Repurposing of MMP inhibitors may hold the key to shortening TB treatments and combating the emergence of MDR strains.

## Introduction

*Mycobacterium tuberculosis* (Mtb) continues to pose a threat to global health. In 2015, 10.4 million people were estimated to have become infected with Mtb and 1.8 million people died because of TB (0.4 million deaths within from TB/HIV co-infection), making Mtb the leading cause of death worldwide from a single infectious agent, ranking above HIV/AIDS[1–3]. TB/HIV co-infection is responsible for about one fourth of all TB deaths and one third of all HIV/AIDS deaths[1, 4]. Furthermore, the incidence of drug resistant TB increased significantly in 2015 compared to previous years[1–3]. Development of new or re-purposed drugs for TB treatment is needed to accomplish the Sustainable Development Goals, which aims to reduce 90% of TB incidence rate by 2030 [1, 5].

Mtb’s success as a pathogen depends upon its ability to reprogram its host environment at both the cellular and tissue levels [6, 7]. The tuberculosis granuloma is characterized by extensive tissue remodeling, extracellular matrix (ECM) deposition and angiogenesis, and ultimately tissue destruction in those granulomas progressing to active disease[8]. The matrix metalloproteinase (MMP) enzymes are major contributors to this remodeling process due to their ability to degrade ECM such as collagen and proteoglycans[9–11]. Among the MMP family, MMP-2 and MMP-9 are known to degrade type IV collagen, fibronectin and elastin in the lung[10, 12, 13], and are markedly up-regulated in expression in human tuberculosis granulomas[14, 15]. Other MMPs have been studied in human tuberculosis tissue and the expression of MMP-1[16–18], MMP-8[19] and MMP-14[20] are significantly up-regulated. Many studies suggested that this up-regulation of MMPs is induced by Mtb infection, and eventually leads to collagen destruction and granuloma necrosis[16–25]. Studies using MMP inhibitors in Mtb infected animal models have generated conflicting data. Hernandez-Pando et al. observed a type-2 cytokine response profile and a delayed granuloma formation in murine pulmonary tuberculosis after treatment with MMP inhibitors[26]. In contrast, Izzo et al. observed increased collagen deposition in early granuloma formation after MMP inhibition, as well as a reduced bacterial burden in the lung at early phase[27]. However, a subsequent study from the same group did not observe a reduced bacterial burden in the lung following MMP inhibition[10]. These studies argue that there is value in further analysis of the impact of MMP inhibition on disease progression and on granuloma architecture.

Most current TB regimens involve a combination of the four drugs (isoniazid, rifampicin, ethambutol, pyrazinamide) as the first-line of treatment. However, the duration of treatment required to generate an enduring cure is usually 6–9 months. Not surprisingly, issues of non-compliance and failure occur frequently, and lead to the ongoing emergence of drug-resistant strains. Selection for drug resistant Mtb happens independently at multiple different geographic locations and is a widespread problem. Therefore, effective strategies to shorten the treatment duration and reduce the incidence of drug resistance are critically important.

In this study, we examined existing human TB granuloma datasets in combination with infectious and non-infectious granuloma models to probe the increased expression of MMP-2 and MMP-9 in Mtb granulomas. Treatment of Mtb-infected mice with a panel of small molecule MMP inhibitors alone had no effect on bacterial burden, but markedly enhanced bacterial killing by the frontline TB drugs INH and RIF approximately 10-fold. We verified the *in vivo* activity of these inhibitors through demonstrating that they block MMP-mediated cleavage of collagen and the mannose binding lectin (MBL). Treatment with these inhibitors also impacted granuloma morphology and appeared to stabilize the blood vessels that irrigated the infection site. Consistent with the improved blood vessel health, we found that MMP inhibition enhanced drug penetrance/retention in the infected tissue, which explains the enhanced efficacy of the anti-TB compounds used in combination with MMP inhibitors.

## Results

### Induction of expression of MMPs in non-infectious and infectious TB granuloma models

**Fig 1:**
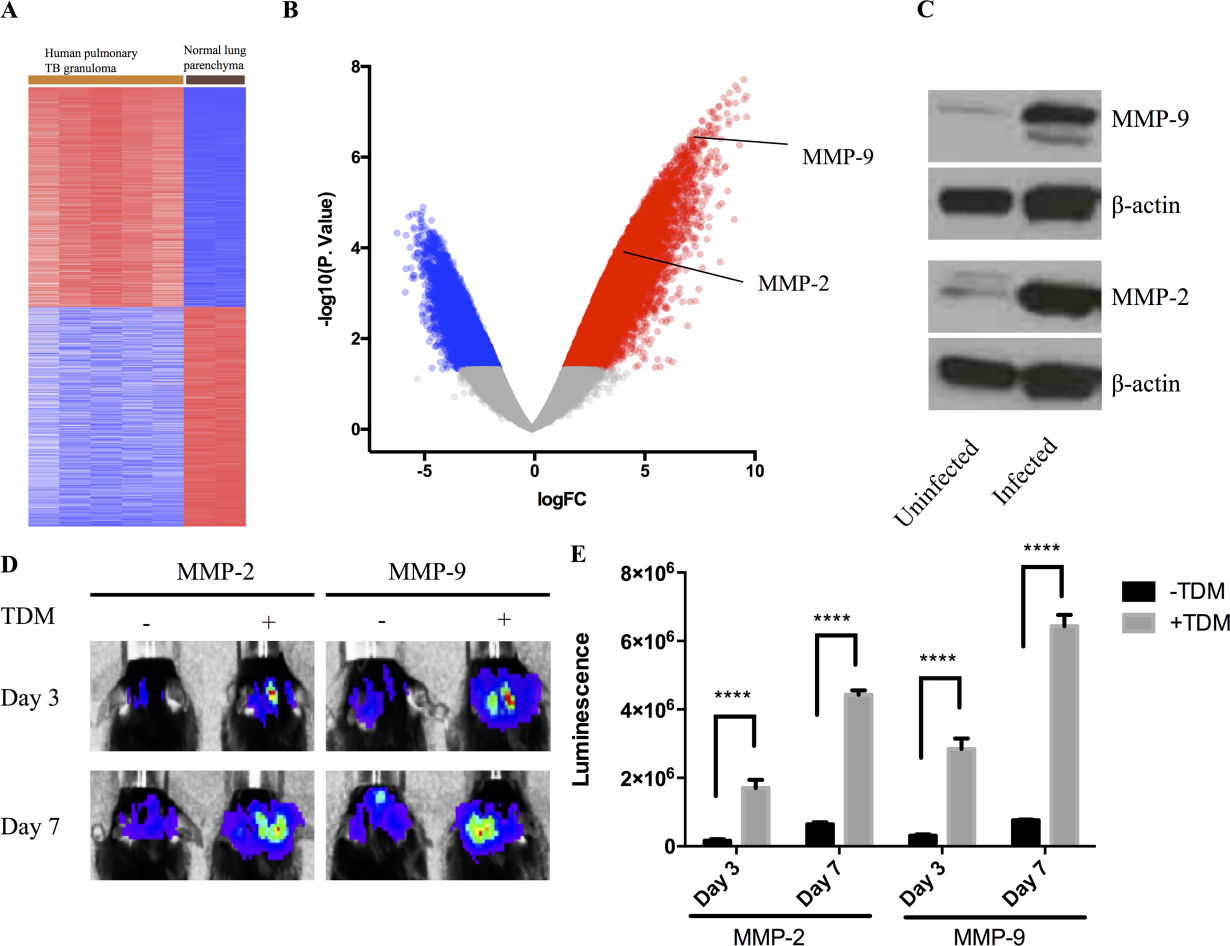
MMP-9 and MMP-2 level are up-regulated during Mtb infection or TDM induction. **(A):** Heatmap showing gene expression profile generated from microarray dataset [34] analysis comparing 5 caseous human pulmonary TB granuloma and 2 normal lung parenchyma (GSE20050). **(B):** Volcano plot showed 12878 up-regulated genes (red dots, p.value < 0.05, Fold change >2) and 8062 down-regulated genes (blue dots, p.value < 0.05, Fold change <0.5) in TB granuloma. MMP-9 and MMP-2 were labeled among the up-regulated genes. **(C):** Protein level of MMP-9 and MMP-2 in uninfected and infected mice lung (n = 3). Representative images were showed. (**D):** Representative images of luminescence from MMP-9 or MMP-2 reporter cell lines within TDM granulomas in C57BL/6J mice. The same mice were imaged at Day 3 and Day 7 (n = 5). Experiment performed twice with similar result. (**E):** Quantitative analysis of luminescent signal at Day 3 and Day 7 of MMP-2 and MMP-9 reporter cell lines within TDM granuloma (n = 5). Data represented mean ± SD. ****: p < 0.0001, 2-way ANOVA with Šidák multiple comparison test.

Previously we had performed transcriptional analysis on material acquired from cryosections from human pulmonary tuberculosis granulomas[14]. We re-analyzed the datasets from 5 caseous human pulmonary TB granuloma and 2 normal lung parenchyma (GSE20050)[14]. Differential analysis was performed using GEO2R to investigate the differential transcriptomic signature in TB granuloma tissue compared to the uninvolved tissue (Fig 1A). Among the differentially-expressed genes, transcripts for both MMP-9 and MMP-2 were significantly more abundant, over 190 fold (log_2_(FC) = 7.6, p value, adjust p value < 0.001), and over 40 fold (log_2_(FC) = 5.35, p value = 0.001, adjusted p value = 0.016), respectively (Fig 1B). This is consistent with previous measurements of MMP expression in human tuberculosis granulomas[11, 16–21, 23, 24].

To examine the induction and expression of MMP-2 and MMP-9 in experimental infectious and non-infectious murine TB granuloma models we used both a subcutaneous granuloma model[14, 28, 29] for *in vivo* imaging in parallel with the more conventional, intranasal Mtb challenge. Western blot analysis of equivalent amounts of tissue from Mtb-infected and uninfected mouse lungs confirmed the increased expression of MMP-2 and MMP-9 in the Mtb-infected tissue (Fig 1C). In order to visualize the up-regulation in transcription of MMP-2 and MMP-9 genes we used reporter RAW cell lines that expressed luciferase under regulation of the MMP-2 and MMP-9 promoter regions. These cells were mixed with a Matrigel suspension containing polystyrene beads coated with the mycobacterial lipid TDM. TDM, or cord factor, is known to have granuloma-inducing properties and has been used previously in non-infectious granuloma models[28, 30–32]. The suspension was inoculated subcutaneously into the mouse scruff, and the tissue response to the challenge was imaged at days 3 and 7 post-inoculation. Quantification of the level of luciferase expression (Fig 1D and 1E) demonstrated the up-regulation of MMP promoter activity in the TDM-bead containing granulomas in comparison to those containing uncoated beads. These data confirm previous reports of up-regulated expression on MMPs in both human TB granulomas and the murine experimental granuloma models used in the current study[16–25].

### The MMP inhibitor Marimastat impacts granuloma architecture and increases the potency of INH

**Fig 2:**
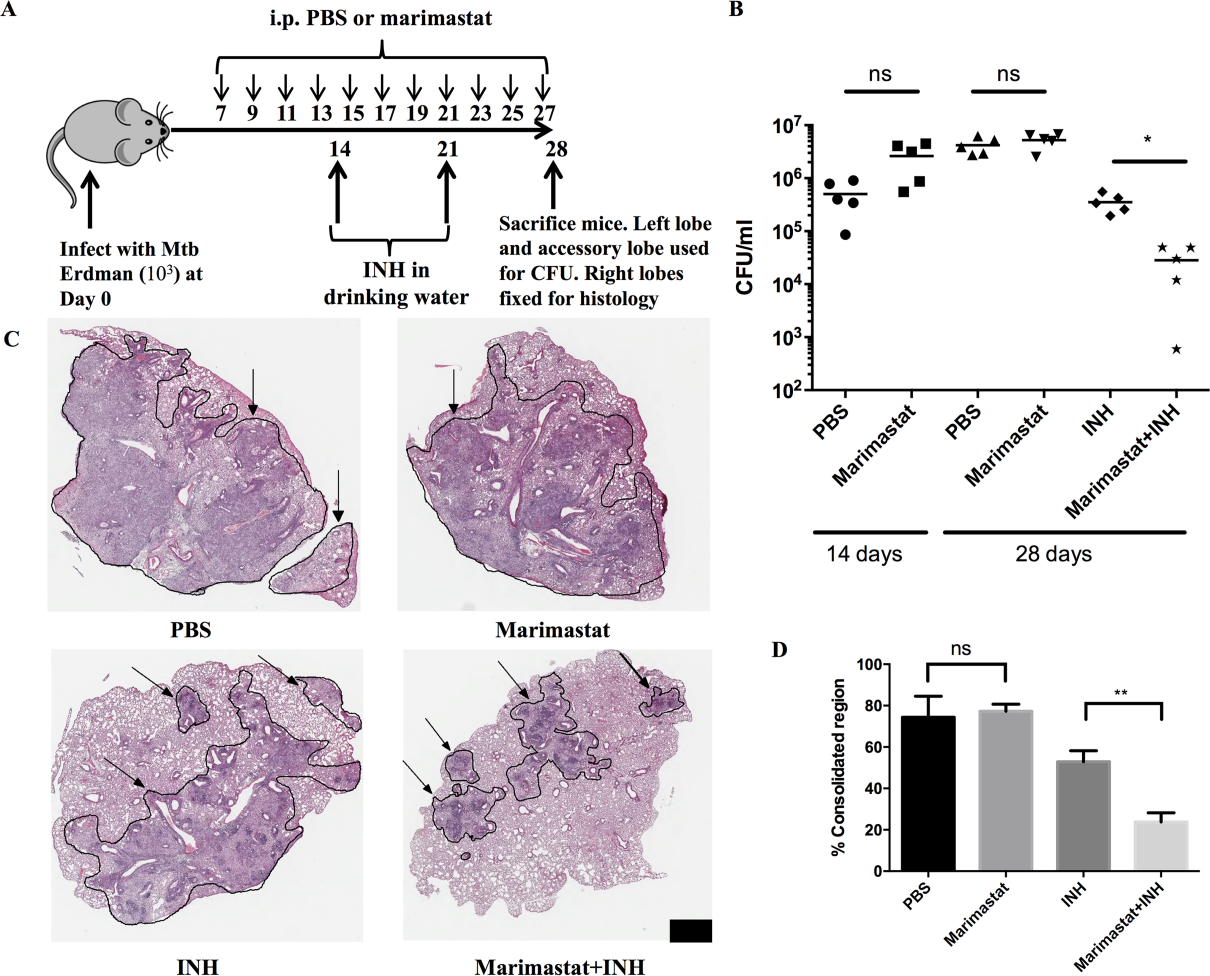
MMP inhibitors facilitate INH killing of Mtb. **(A):** Experimental setting of Marimastat’s effect in lung infection model (n = 5). (**B):** CFU count of 2 groups (PBS and Marimastat) at Day 14, and 4 groups (PBS, Marimastat, INH and Mariamstat+INH) at Day 28 post infection. Each dot represented one mouse (n = 5). Experiment was repeated 3 times with similar observation. Data showed results from one representative experiment. **(C):** H&E stain of the 4 groups (PBS, Marimastat, INH and Mariamstat+INH) at Day 28 infection (n = 5). Scale bar: 1mm. Arrow indicated consolidated region circled by black line. **(D):** Quantification of inflammatory region percentage within tissue of in all mice from different treatment groups (n = 5). Data represented mean ± SD. **: p < 0.01, One-way ANOVA with Šidák multiple comparison test.

Marimastat is a broad spectrum MMP inhibitor that was developed as an anti-neoplastic drug candidate[33–35]. Treatment of mice with the drug induced morphological alterations in both subcutaneous TB granuloma models, including the TDM-bead Matrigel model and the Mtb-Matrigel subcutaneous challenge model (S1 Fig). The treatment appeared to result in higher cellular consolidation within the matrix. We probed the biological significance of this morphological change to disease progression through determination of both bacterial burden and histological change in mice challenged intranasally with Mtb. The design of the experiment is shown in Fig 2A. Mice were infected with Mtb then treated with PBS (control) or Marimastat from day 7 onwards. Mice were also treated with the frontline TB drug INH (± Marimastat) to explore the potential impact of MMP inhibition on drug efficacy. Mice were sacrificed at day 28 and process for both histology and bacterial counts.

There was no statistically significant difference in bacterial load between the Marimastat and PBS groups at Day 14, Day 28 or Day 42 (Figs 2B and S2), consistent with previous reports that inhibition of MMP activity did not impact bacterial burden[10, 26]. However, when INH was added into the drinking water, a synergistic effect was observed in the INH and Marimastat co-treated group (Fig 2B). INH alone reduced the bacteria load approximately ten-fold compared to the PBS control, while Marimastat and INH in combination reduced the bacterial load by another log over the INH only group (Fig 2B). This synergistic effect was significantly reduced but still observable by Day 42 (S2 Fig). The combination treatment also reduced size of the regions of cellular consolidation within the infected lung tissue, as shown by H&E staining (Fig 2C). This reduction is likely due to both MMP inhibition and the reduced inflammatory stimulation caused by the lower number of bacteria. The percentages of consolidated region within the whole tissue were measured and the percentages of consolidated region in PBS and Marimastat groups were comparable (Fig 2D). This percentage was decreased in the INH only group, and further reduced in the Marimastat and INH group (Fig 2D). This reduction correlates most closely with the CFU measurements.

These data suggest that targeting MMP activities enhances the efficacy of INH against Mtb. To determine if this was due to activity at the level of the host cell or the host tissue environment we treated Mtb-infected BMDM with Marimastat in the presence and absence of INH. This tissue culture infection model did not recapitulate the synergistic effect of the two drugs combination (S3 Fig), indicating that the synergy observed is dependent on the *in vivo* tissue environment.

### The synergistic activities extend to both RIF and to other MMP inhibitors

**Fig 3:**
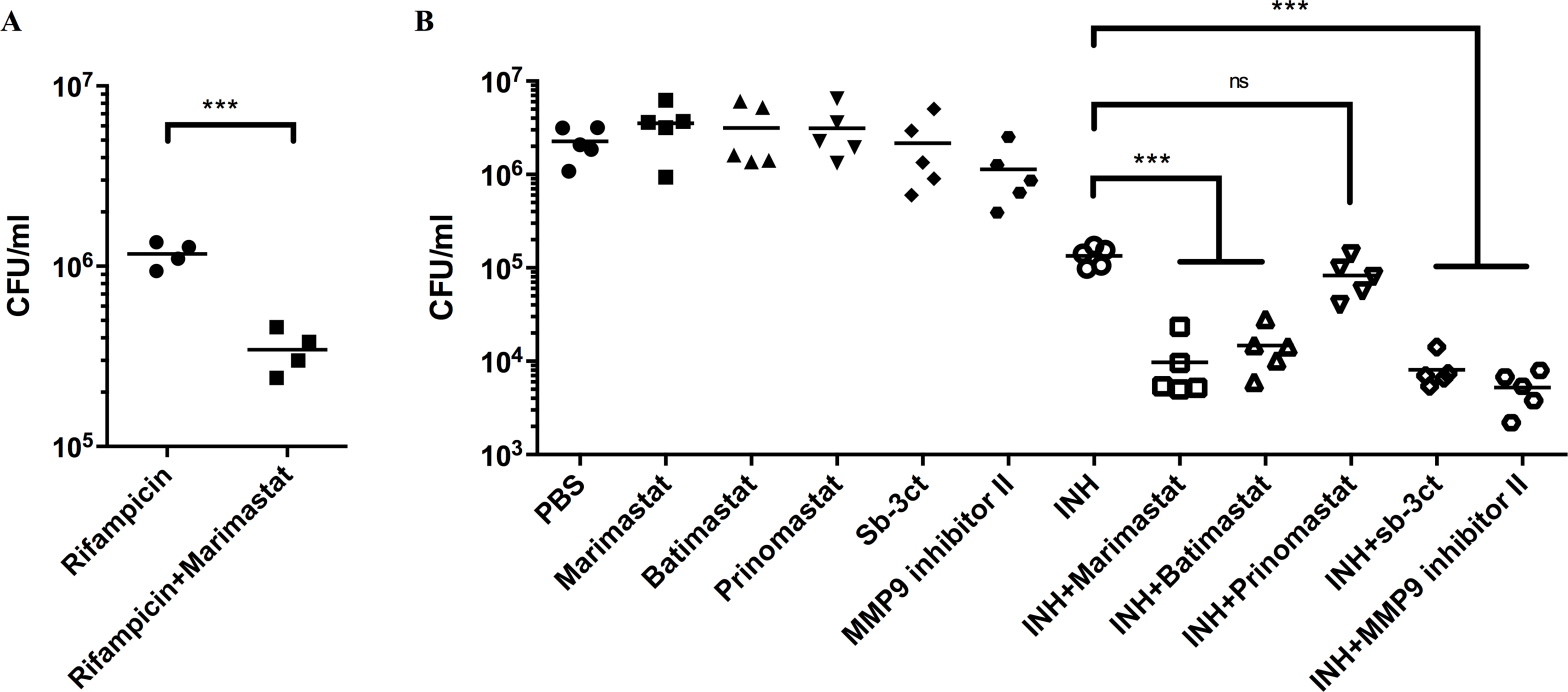
Other frontline TB drug (RIF) and other MMP inhibitors can induce synergistic effect to reduce Mtb burden. **(A):** CFU count of lung tissue from Mtb-infected mice treated with Marimastat and RIF. Each dot represented one mouse (n = 4). Data represented mean. ***: p < 0.001, Two-tailed Unpaired Student *t* test with Welch-correction. **(B):** CFU count of lung tissue from Mtb-infected mice treated with other MMP inhibitors and INH. Each dot represented one mouse (n = 5). Data represented mean. ***: p < 0.001, One-way ANOVA with Šidák multiple comparison test.

Next, we investigated whether this synergetic effect could be observed with a different TB drug, RIF and/or with other MMP inhibitors. The combination of RIF and Marimastat decreased bacterial burden in comparison with RIF alone (Fig 3A), demonstrating that the ability of Marimastat to enhance anti-TB drug activity was not restricted to INH, and therefore unlikely to be linked to the specific mode of action of one anti-TB drug.

We then tested a panel of MMP inhibitors in combination with INH to determine if the synergy was Marimastat-specific. The panel of MMP inhibitors included (1) Batimastat, which shares a similar structure to Marimastat, but has lower water solubility, (2) Prinomastat, a structurally-unrelated broad spectrum MMP inhibitor, (3) Sb-3ct, a specific inhibitor of MMP-9 and MMP-2 and (4) MMP-9 inhibitor I, an inhibitor that exhibits much more specific activity against MMP-9[33]. In isolation, all these MMP inhibitors had minimal effect on bacterial burden. However, with the exception of Prinomastat, all the MMP inhibitors enhanced bacterial killing by INH (Fig 3B). Therefore, the synergistic effect between MMP inhibition and antibiotics is not limited to Marimastat and INH, but applied to other MMP inhibitors and other frontline TB drugs. Furthermore, this synergistic effect is likely due to inhibition of MMP-9/MMP-2.

### Inhibition of MMP results in an increase in both collagen and mannose binding lectin (MBL) within the infected tissue

**Fig 4:**
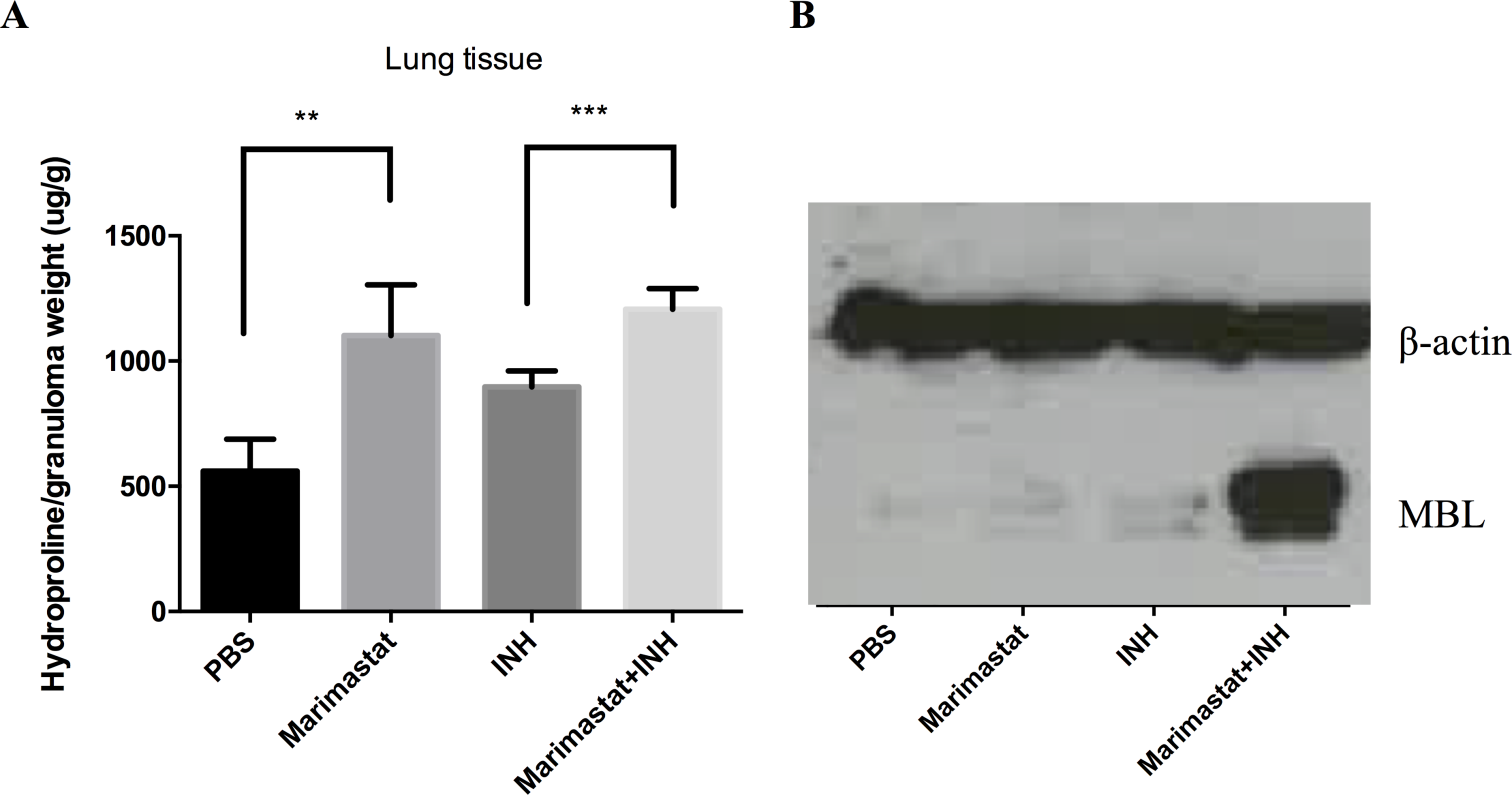
Inhibition of MMP results in an increase in both collagen and mannose binding lectin (MBL) within the infected tissue. **(A):** Hydroxyproline concentration in lung tissue from infected mice under different treatments (n = 5). Data represented mean ± SD. **: p < 0.01, ***: p < 0.001, One-way ANOVA with Šidák multiple comparison test. (**B):** MBL protein level from lung lysate of infected mice with different treatments (n = 5).

MMPs within the tissue have a wide range of substrates[12, 27, 34–37]. The most obvious target of these proteases is the extracellular matrix, including collagen. To verify the biological activity of Marimastat within the infection site we measured the levels of substrate within the tissue. The level of hydroxyproline, a modified amino acid specifically released from collagen degradation, was measured from infected mouse lung tissue. In the groups with Marimastat we observed a higher level of hydroxyproline, indicating higher collagen concentration (Fig 4A). Similar data were also generated in the TDM-Matrigel and the Mtb-Matrigel granuloma models (S4 Fig). However, interpretation of these data is complex because the fibrotic response itself is biologically-active[28, 29] and the outcome, greater collagen deposition, could be generated by other mechanisms in addition to reduced degradation of collagen by MMPs.

Other known MMP substrates include the mannose-binding lectin (MBL), which has a collagen-like domain that is cleaved by MMP-2, MMP-9, and MMP-14[38]. MBL is an interesting immune modulator because it can recognize mycobacterial surface lipidoglycans[39–41], act as an opsonin, and activate the complement cascade[41, 42]. We examined the levels of MBL in the lung of Marimastat and INH treated, infected mice. Marimastat and INH combined treatment resulted in the highest MBL level (Figs 4B and S5A). The MBL level was relatively low in the Marimastat group compared to the Marimastat and INH group, which was due to higher MMP-2 and MMP-9 protein levels in the Marimastat group (S5B Fig). This may be a consequence of the larger number of bacteria present in the Marimastat group (Fig 2B), which could lead to MMP overproduction to compensate for the inhibition of Marimastat.

### Increased blood vessel pericyte coverage by Marimastat treatment

**Fig 5:**
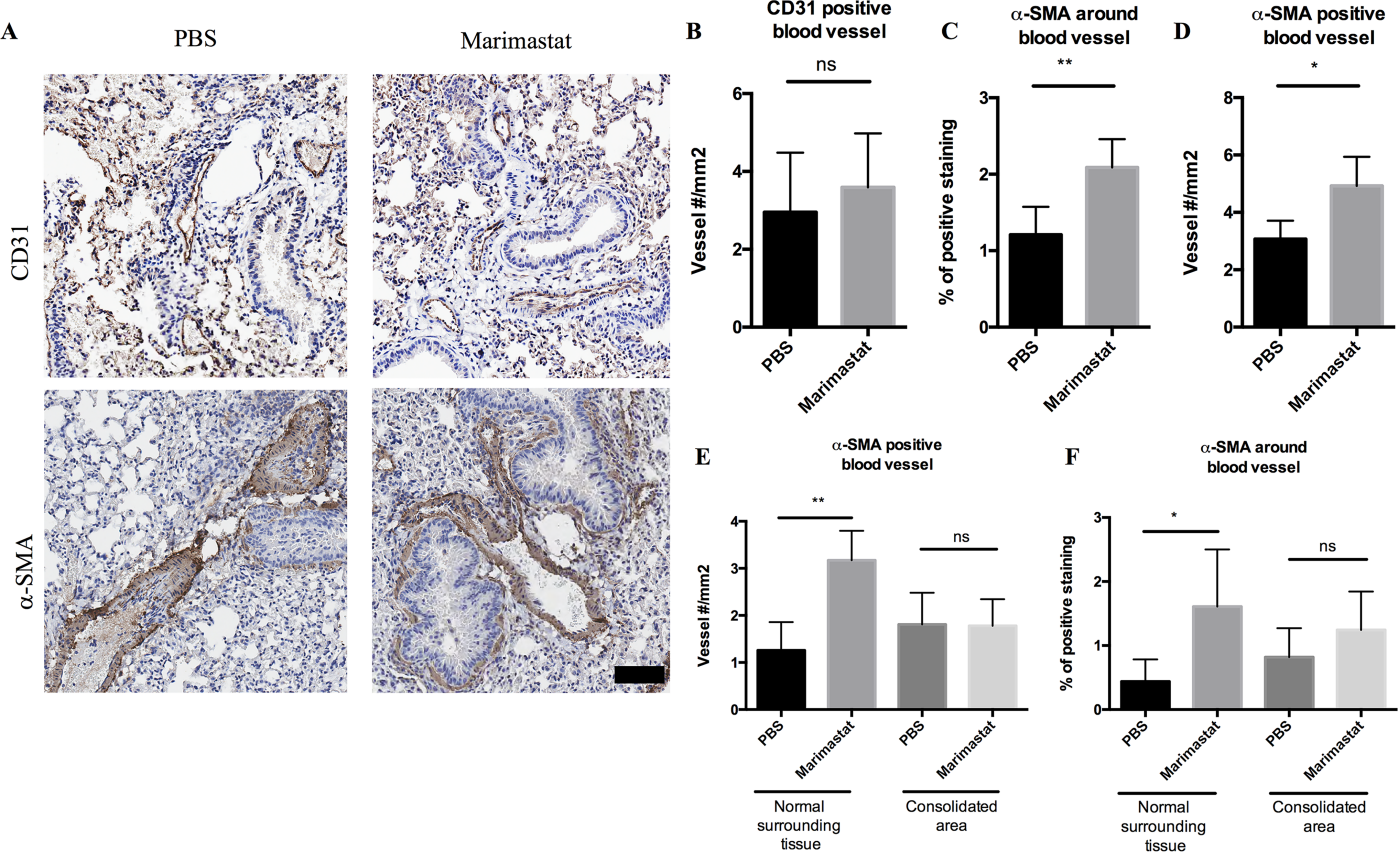
CD31 and α-SMA staining in the lung of infected mice treated with Marimastat. **(A):** Mice infected with Mtb were treated with PBS (n = 5) or Marimastat (n = 5). Lung tissue from infected mice was stained for CD31 (upper panel) and α-SMA (lower panel). Both PBS and Marimastat groups had positive staining of CD31 and α-SMA. Scale bar: 80μm. (**B, C, D):** Quantification of CD31 positive blood vessel number (B), percentage of α-SMA positive staining (C), and α-SMA positive blood vessel number (D) in PBS or Marimastat treated mice (n = 5) 4-week post infection. Data represent mean ± SD. *: p < 0.05, **: p < 0.01, Two-tailed Unpaired Student *t* test with Welch-correction. **(E, F):** Quantification of percentage of α-SMA positive staining (E), and α-SMA positive blood vessel number (F) in consolidated area (granuloma like area) and surrounding normal tissue from PBS or Marimastat treated mice (n = 5) 4-week post infection. Data represented mean ± SD. *: p < 0.05, **: p < 0.01, One-way ANOVA with Šidák multiple comparison test.

There is mounting evidence that MMPs have a strong effect on angiogenesis beyond ECM remodeling—MMPs can release ECM-bound angiogenic factors, detach pericytes from blood vessels, and degrade endothelial cell-cell adhesions[37, 43]. To test whether MMP inhibition impacts the vasculature at the infection site, we stained lung tissue of 4-week infected mice treated with PBS or Marimastat with CD31 for endothelial cells[44, 45]. We observed positive CD31 staining in sections from both PBS and Marimastat groups (Fig 5A, upper panel). Blinded histological analysis indicated that there was no significant difference in the number of CD31 positive blood vessels upon Marimastat treatment (Fig 5B).

We also stained tissue with alpha smooth muscle actin (α-SMA). α-SMA stains for pericytes which wrap around the endothelial layer to supply nutrients[44, 46], as well as for alpha smooth muscle cells around the bronchus, which can be easily distinguished from blood vessels by morphology the bronchus has a single layer of columnar epithelial cells). Both the PBS group and Marimastat group had positive staining of α-SMA (Fig 5A, lower panel). Blinded histological analysis indicated that the percentage of positive α-SMA area was significantly higher in Marimastat group than that in PBS group (Fig 5C). Based on the positive staining of α-SMA, the blood vessel numbers from both groups were counted. The Marimastat group had significantly more α-SMA positive blood vessels than the PBS group (Fig 5D). This suggested that treatment with Marimastat increased the number of blood vessels covered by pericytes, while the total blood vessel number remained unchanged. Moreover, we counted the α-SMA positive blood vessels in the consolidated area (granuloma like structure) and the normal surrounding area separately. We found that the increase of pericyte coverage occurred in the surrounding area instead of in the consolidated area (Fig 5E and 5F). The numbers of blood vessels with positive α-SMA staining were not significantly different between the PBS and Marimastat treated groups at 2 weeks post infection (S6 Fig). As infection progressed to 4 weeks, the number of α-SMA positive blood vessels remained similar in the PBS control group, but was significantly increased by Marimastat treatment (S6 Fig), indicating MMP inhibition increases pericyte-covered blood vessels over time.

In summary, MMP inhibition reduced blood vessel abnormality by increasing pericyte coverage around the blood vessels. This increase of pericyte-covered blood vessel number and the improvement of blood vessel health could potentially enhance drug (such as INH) delivery or retention in the infected tissue environment.

### Marimastat treatment reduces blood vessel leakage and increases drug delivery/retention in the lung

**Fig 6:**
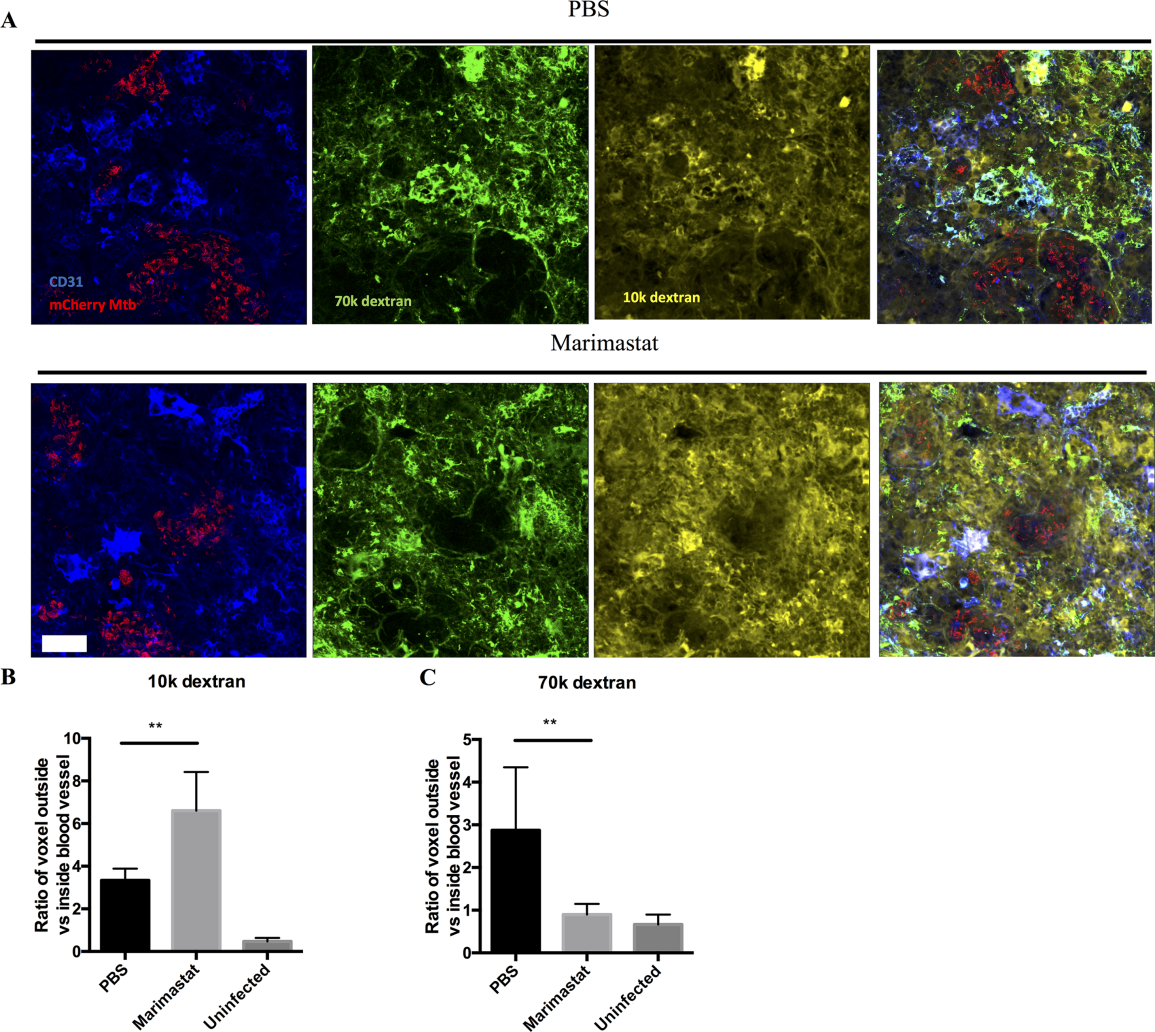
Leakage and delivery of blood vessels measured by fluorescent dextran. **(A):** Mice infected with mCherry Mtb bacteria (red) were treated with PBS (n = 3) or Marimastat (n = 3). 70k dextran (green) and 10k dextran (yellow) were injected to mice before euthanization. Lung tissue from infected mice was stained for CD31 (blue) to label the blood vessels. Both PBS and Marimastat groups had positive staining of CD31 and positive signal of 70k and 10k dextrans. Scale bar: 20μm. (**B):** Quantification of the ratio of 10k dextran outside vs inside of blood vessel (**C):** Quantification of the ratio of 70k dextran outside vs inside of blood vessel. Data represented mean ± SD. **: p < 0.01, One-way ANOVA with Šidák multiple comparison test.

Inflammation is known to increase vascular permeability[47–49] and we hypothesized that the increased pericyte coverage induced by Marimastat treatment might reflect a reversal of this permeability. To test the impact of Marimastat treatment on vascular permeability, we injected fluorescent dextran with different molecular weights to Mtb infected mice. Under normal, homeostatic conditions one would expect 10kDa dextran to passively diffuse out of the vasculature, while 70kDa dextran would be retained for longer. However, under inflammatory conditions, one would expect to see increased leakage of the 70kDa dextran[47]. Lung tissues from treated mice, infected or uninfected with Mtb, were fixed and stained for CD31 to mark the blood vessels, and imaged by a confocal microscope. Both PBS group and Marimastat group from infected mice showed positive staining of CD31 and fluorescent signal from the two dextran dyes (Fig 6A). We scored the 10kDa and 70kDa dextran signal that either co-localized with the CD31-positive blood vessels, or was present in the tissue outside the CD31-positive regions (Fig 6B and 6C). Compared to uninfected samples, Mtb-infected tissue had more 10kDa dextran outside blood vessels, indicating Mtb infection promotes neo-vascularization (Fig 6B). These new blood vessels appeared to be leaky, as the levels of 70kDa dextran outside blood vessels were much higher in infected mice (treated with PBS) relative to the uninfected group (Fig 6C). Marimastat treatment reduced 70kDa dextran to a level similar to that of uninfected animals (Fig 6C), while further increased the 10kDa dextran level (Fig 6B). This suggests that Marimastat reduced blood vessel leakage and increased small molecule delivery in the lung, consistent with a “normalization” of the vasculature.

**Fig 7:**
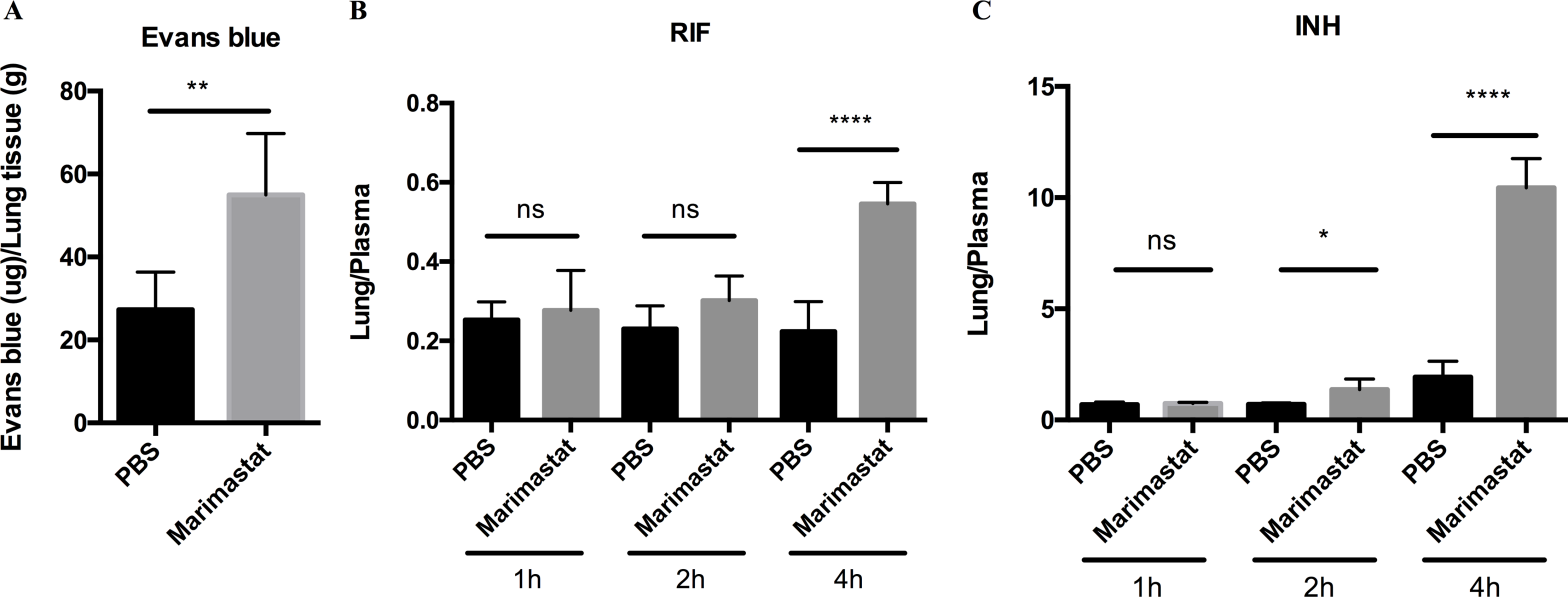
Delivery and/or retention of Evans blue dye or frontline TB drugs in infected animals treated with Marimastat. (**A**): Mtb infected mice treated with or without Marimastat were injected intravenously with Evans blue dye before euthanization. Retention of Evans blue dye within the lung of infected mice treated with or without Marimastat. Data represented mean ± SD. **: p < 0.01, Two-tailed Unpaired Student *t* test with Welch-correction. Experiment was repeated twice. (**B** and **C**): Mtb infected mice treated with or without Marimastat were injected intravenously with RIF and INH before euthanization. At indicated time points, lung and blood samples were collected for drug measurement. The concentration of RIF (**B**) and INH (**C**) in the lung were normalized by drug concentration in the plasma. Data represented mean ± SD. *: p < 0.05, ****: p < 0.0001, One-way ANOVA with Šidák multiple comparison test.

The normalization of the vasculature in Mtb granulomas has been shown to improve small molecular delivery[44]. To determine whether or not Marimastat treatment could have a similar impact, we injected Evans blue dye intravenously into infected mice treated with or without Marimastat, and measured the dye retention in the lung tissue. We observed that there was an increase in Evans blue dye in the lungs of mice treated with Marimastat (Fig 7A), indicating that inhibition of MMP activity enhanced small molecule delivery and/or retention in the infected tissues. To further demonstrate this enhanced delivery and/or retention by MMP inhibition also applies to frontline TB drugs, we injected RIF and INH intravenously into infected mice treated with or without Marimastat. Intravenous injection of drugs minimizes inter-animal variation in absorption, which is a frequent confounding factor, particularly for RIF. Drug concentrations were measured in the harvested lung tissues at different time points by high-pressure liquid chromatography coupled to tandem mass spectrometry (HPLC-MS/MS) analysis, and normalized to the drug concentrations in the plasma [50]. 4h post drug injection, the Marimastat-treated group had significantly higher RIF and INH lung/plasma ratios than the PBS control group (Fig 7B and 7C), suggesting an enhanced drug delivery/retention by MMP inhibition. Although the drug concentrations decreased in the lung and plasma over time (S7 Fig), MMP inhibition maintained a relatively high INH concentration in the lung (S7C Fig). RIF concentration was not increased in the lung but decreased in the plasma upon MMP inhibition, suggesting a faster turn-over rate of RIF. Taken together, these data indicated that inhibition of MMP activity enhanced frontline TB drug delivery and/or retention in the infected tissues through improving blood vessel integrity.

## Discussion

Current therapeutic regimens for tuberculosis are cumbersome because of the need for multiple drugs (3–4) that have to be taken for 6–9 months. This places considerable strain on many healthcare systems, particularly those in under-resourced settings, and leads to ongoing problems of non-compliance and the emergence of drug-resistant Mtb strains. Given these challenges any strategy to increase the potency of our current anti-TB drugs could have tremendous benefit on tuberculosis control programs across the world. Here we show that small molecule MMP inhibitors increase the killing activity of the frontline anti-TB drugs INH and RIF. This enhanced killing was observed for several different MMP inhibitors suggesting that it is their anti-matrix metalloproteinase activity that confers their synergistic activity. We found that MMP inhibition improved blood vessel health, leading to increase of drug delivery and/or retention in the lung, which resulted in increased drug efficacy.

The majority of Mtb animal studies use the mouse as host. Different mouse strains and different infection methods created a number of Mtb murine models [51, 52]. However, mice infected with Mtb fail to form the well-defined and highly stratified granuloma structure, which is commonly seen in human TB patients [6, 7]. The granuloma structure developed in our murine model resembles an early stage human granuloma, where activated macrophages surround the infected cells with a layer of lymphocytes at the peripheral [51, 52]. Murine granulomas rarely progress to necrosis, which characterize late stage or disseminated human TB granulomas [6, 7]. Moreover, mice do not express MMP-1, which, along with other MMPs, is associated with tissue destruction and transmission in disseminated human granuloma [23]. However, while there are limitations to murine models of Mtb pathogenesis, one cannot ignore the convenience of such a tractable experimental system if used appropriately. Different murine models are widely used to study essential bacterial genes or host immune response, because of their availability and well-characterized genetic variations. Recently, studies using resistant mouse strain infected with *M*. *marinum* [53], or susceptible mouse strain infected with Mtb [51, 52] observed granuloma-like structures with necrotic center in the lung, resembling the human granuloma. Moreover, murine models provide an *in vivo* platform to screen novel TB drugs for efficacy or synergy with frontline TB drugs, before advancing to costly non-human primate experiments or human clinical trials. We therefore believe that the mouse represents a valuable tool for discovery prior to downstream validation of ones finding in more restrictive platforms.

Treatment of Mtb-infected mice with Marimastat reduces extracellular matrix turnover and breakdown of the mannose-binding protein MBL. Histological analysis and vasculature-permeability experiments both indicate that the blood vessels in the TB granulomas were stabilized by Marimastat treatment. Healthy blood vessels, instead of leaky vessels, can improve the amount of drug, that is given orally, delivered to the lung. As a result, there is an improved tissue retention of small molecules and anti-TB drugs. Moreover, healthy blood vessels can potentially enhance drug accessibility to the bacteria at the center of a confined structure. Improvement of drug delivery through the normalization of blood vessels is widely accepted as a means of enhancing the efficacy of anti-cancer drugs[54, 55]. Several phase III clinical studies have shown that combination of conventional chemotherapeutic drugs with FDA approved anti-angiogenesis drugs can significantly improve survival of patients with non-small lung cancer[56], breast cancer[57] and metastatic colorectal cancer[58, 59]. Moreover, the anti-VEGF drug, Bevacizumab, which is approved by FDA to treat metastatic colorectal cancer[60], also enhances small molecule delivery to tuberculosis granulomas in rabbits[44]. Additionally, Oehlers et al. showed that VEGF inhibitors can synergize with RIF to reduce bacterial burden of *M*. *marinum* in Zebrafish [61]. Based on our findings, it is reasonable to add doxycycline, an anti-mycobacteria antibiotic [62] and the only FDA-approved MMP inhibitor, to the current anti-TB drug regimens as an adjunctive drug. Specific MMP inhibitors like Marimastat were well-tolerated in animals and have been tested in clinical trials to target cancer metastasis [33, 63]. Respiratory dysfunction, a possible adverse effect considering increased fibrosis by MMP inhibition in the lung, was not identified in these clinical trials. Moreover, these side effects may be prevented by avoiding high-dose treatment [33, 63].

These data underline the importance of exploiting strategies that improve the efficacy of existing drugs as a readily tractable means of increasing the effectiveness of our anti-TB therapy. The possible addition of cheap, well-tolerated drugs such as MMP inhibitors to current multi-drug regimens is a practical and attractive means of increasing potency.

## Methods

### Ethics statement

All animal experiments were performed in strict accordance with the National Institutes of Health “Guide for the Care and Use of Laboratory Animals”, and approved by Cornell University Institutional Animal Care and Use Committee under protocols 2006–0019, 2011–0086, 2010–0100, 2013–0003. All animal experiments performed inside Biosafety Level 3 facility were approved under protocol 2011–0086. All efforts were made to minimize suffering.

### Mouse and Bone marrow-derived macrophage

C57BL/6J mice, MBL knockout mice (B6.129S4-Mbl1^tm1Kata^ Mbl2^tm1Kata^/J) were obtained from the Jackson Laboratory and housed under pathogen-free conditions.

Bone marrow-derived macrophages (BMDMs) were isolated from bone marrow of C57BL/6J wild type mice, and maintained in DMEM (Corning Cellgro) containing 10% FBS (Thermo Scientific), 10% L929-cell conditioned media, 2mM L-glutamine, 1mM sodium pyruvate and antibiotics (penicillin/streptomycin) (Corning cellgro), at 37^°^C in a 5% CO2 incubator[64, 65].

### Reporter cell line, plasmid construction, IVIS imaging

To validate the up-regulation of MMP-2 and MMP-9 during Mtb infection, we constructed reporter cell lines that have promoters of *Mmp-2* and *Mmp-9* upstream of GFP and luciferase encoding cassettes. The luminescent signal was detected and quantified by the IVIS imaging instrument. The primers sequences for *Mmp-2* and *Mmp-9* promoter’s region were designed using PrimerPremier5 (Table 1).

**Table 1:** Primer sequence of *Mmp-2* and *Mmp-9*.

	Primer sequence
*Mmp-2*	F: CCATCGATGCAAAGGTGACAACCGTGA
	R: GGACTAGTGGCTGGAAGAGTGCTGGC
*Mmp-9*	F: CCATCGATTAGAAGCAGGAGGACCCGA
	R: GGACTAGTTGGCTAACGCTGCCTTTG

Mouse genome DNA was used as template to amplify the sequences, which were inserted into plasmid pGreen-Fire. Vectors with genes of interest were transferred into RAW 264.7 macrophage cell line (ATCC TIB-71) using a lentivirus infection system. Single colonies were picked and validated for expression levels of GFP and luciferase. We used a non-infectious Mtb trehalose dimycolate (TDM) granuloma model to test whether these stable cell lines can express GFP and luciferase [29, 31]. TDM coated beads were suspended in Matrigel, mixed with the reporter RAW cell lines, and inoculated subcutaneously in the scruff of a mouse. The mice were anesthetized at certain time points, injected with luciferase substrate and imaged with a IVIS machine (Caliper Lifescience).

### TDM granuloma model and the Mtb/Matrigel granuloma model

TDM matrigel injection method has been described previously[14, 28, 29]. Briefly, 1mg TDM (Enzo life science) was dissolved in 100μl chloroform/methanol mixture (2:1). 4μl of the dissolved TDM (40μg) was transferred to a glass tube and dried under nitrogen gas. 150μl polystyrene microspheres (approximately 10^4^ particles 79.4±0.5μm Duke Scientific) were washed with 1ml PBS and added to the tube. The tube was sonicated in 55°C water bath for 1h, in order to coat the TDM onto the beads. 5×10^6^ BMDMs or reporter RAW cells were harvested and mixed with TDM-coated beads in 400μl cold Matrigel (Corning). 27G syringe was used to inject the mixture subcutaneously in the mouse scruff. After 7 days, mice were sacrificed and matrigel tissue was extracted for histology or collagen content measurement.

For Mtb/Matrigel granulomas, Mtb Erdman from frozen titered stocks was passaged through 25G syringe 8 times to dissociate clumps. The stock was diluted 1000 times in PBS containing 0.05% Tween 80 and 10^3^ bacteria in 25μl were mixed with 400μl matrigel containing 5×10^6^ BMDMs, and injected subcutaneously into the mouse scruff. After 28 days, mice were sacrificed and matrigel tissue was extracted for histology or collagen content measurement.

### Lung infection and drug treatment

In order to investigate drug effects on pulmonary Mtb infection, we infected mice intranasally as described previously[66, 67]. Mice were anesthetized with isoflurane and then 25μl of PBS+0.5% Tween 80 containing 10^3^ bacteria was delivered into the nares of the mice. Erdman WT and the fluorescent reporter strains Erdman (*smyc’*::mCherry) were used for infection experiments as described previously[66]. After mice were euthanized, the left lobe and the accessory lobe were used for CFU plating, while the right lobes were either fixed in 4% paraformaldehyde for confocal microscopy or histological analysis, or used for protein extraction and collagen content measurement.

Marimastat and other MMP inhibitors were delivered via intra-peritoneal injection every other day starting 7 days post infection. INH (12.5mg/kg/day) and RIF (5mg/kg/day) were added to the drinking water starting 14 days post infection and replenished every week.

### Tissue collagen quantitation

Method is adapted from C. Kliment et al[68]. Briefly, tissue samples were weighed and put into glass tubes, which were placed in 100°C heat blocks inside a fume hood until the tissue were completely dry[68]. 2ml of 6M HCl was added to each tube, which was sealed under inert gas and incubated on the heat block for 24h. 2ml of PBS was added to each tube to reconstitute the sample and incubated at 60°C for 1h. The samples were centrifuged at 14,000rpm to remove the undissolved substance and analyzed for hydroxyproline content. 400μl sample was incubated with 200μl chloramine-T solution (50mM chloramine-T, 30% v/v 2-methoxyethnol and 50% v/v hydroxyproline buffer) for 20 min at room temperature. Then 200μl perchloric acid (BioVision) was added and tubes were incubated 5min at room temperature. Finally, 200μl p-dimethylamino-benzaldehyde solution (1.34M p- dimethylamino-benzaldehyde dissolved in 2-methoxyethnol) was added to each tube and tubes were incubated at 60°C for 20min. Absorbance measurements were read in a 96-well plate at 557nm on Envision plate reader (PerkinElmer).

### Confocal immunofluorescence microscopy

Confocal analysis was conducted as detailed previously[66]. Briefly, lung tissue was sectioned in to 1 mm thick slices with a razor blade. Then tissue was blocked and permeabilized in PBS + 3% BSA + 0.1% Triton X-100 at room temperature for 1h in the dark. Samples were incubated with primary antibody (CD31, 1:100, BD; α-SMA, 1:200, Abcam) overnight at 4°C and corresponding secondary antibodies, in the presence of DAPI (1:500) and Alexa fluor 647 conjugated Phalloidin (1:50) at room temperature for 2h in the dark. Samples were washed 3 times with PBS and mounted with mounting medium (Vectorshield). Imaging was conducted using a Leica SP5 confocal microscope and signal was quantified by Volocity software[66].

### Immunohistochemistry and quantitative image analysis

Infected lung tissues were fixed and sectioned by the Histology lab of Animal Health Diagnostic Center in Cornell University. Briefly, unstained slides were hydrated and stained with primary antibodies (CD31, 1:1000, BD; α-SMA, 1:500, Abcam) overnight at 4°C. Slides were washed and stained with secondary antibodies (1:200, biotinylated goat anti-rabbit IgG antibody, Vector lab) and ABC kit at room temperature for 2h. Sections were developed with DAB and mounted for digital scanning (Scanscope, Aperio Technologies). Blind quantitative analysis of IHC images were performed with the Imagescope (Aperio Technologies) software.

### Western blot

The middle lobe of the right lung from mice infected with Erdman strain Mtb was homogenized within cold RIPA buffer supplemented with protease inhibitor (Roche) to extract protein. Proteins were run on 10% SDS-PAGE gel and later transferred to PVDF membrane (Millipore). The membrane was incubated with primary antibody (MBL 1:100 Hycult; MMP-2 and MMP-9, 1:2000 Abgent) overnight at 4°C and with secondary antibodies at room temperature for 2h. The membranes were incubated with Super Pico Chemiluminescent Solution kit for 5min and developed with Amersham Hyperfilm ECL films (GE Healcare).

### Evans blue dye quantitation in infected mouse lung

Mice infected with Erdman Mtb were treated with Marimastat as described above. At 4 weeks post infection, mice received a retro-orbital injection of 25mg/kg Evans blue dye and sacrificed 30min after injection. The left lobe and accessary lobe were used for CFU plating and the right lobes were homogenized and incubated in formamide at 55°C for 20h to extract the dye. The homogenate was centrifuged and the supernatant was read for absorbance at 620nm and 740nm. The following formula was used to correct for contamination with heme pigment[69–71]:

$$E620_{corrected}=E620_{raw}-\left(1.426\times E740_{raw}+0.030\right)$$

A standard curve was used to calculate the amount of dye in the lung, which was further normalized to tissue weight.

### Frontline TB drugs measurement in infected animals by high-pressure liquid chromatography coupled to tandem mass spectrometry (LC-MS/MS) analysis

Methods were adapted from previous studies measuring ethambutol in pulmonary TB lesion [50]. Briefly, mice were infected with Erdman Mtb and treated with Marimastat as described above. At 4 weeks, infected animals received a single retro-orbital injection of RIF 2mg/kg and INH 5mg/kg, and sacrificed 1h, 2h and 4h after injection. Lung tissue was collected and homogenized by adding 3 parts of PBS buffer. Samples were shaken using a SPEX Sample Prep Geno/Grinder 2010 for 5 minutes at 1500 rpm with steel beads. Blood samples were collected in tubes coated with K_2_EDTA and centrifuged at 4000 rpm for 10 min to collect plasma. Standards and QCs were created by adding 10 μL of spiking stock (neat 1 mg/mL DMSO stocks of RIF, INH, and Acetyl-INH diluted in 50/50 (Acetonitrile/water)) to 90 μL of drug free plasma (Bioreclamation) or control lung tissue homogenate. 20 μL of control, standards, QCs, or study samples were added to 200 μL of Acetonitrile/Methanol 50/50 protein precipitation solvent containing 20 ng/mL RIF-d8, INH-d4, and Ac-INH-d4 (Toronto Research Chemicals). Extracts were vortexed for 5 minutes and centrifuged at 4000 rpm for 5 minutes. 100 μL of supernatant was combined with 100μL of 2% cinnamaldehyde in methanol to derivatize INH. Mixture was vortexed for 30 minutes to complete reaction. 100 μL of mixture was combined with 100 μL of Milli-Q water prior to HPLC-MS/MS analysis.

High-pressure liquid chromatography (HPLC) coupled to tandem mass spectrometry (LC/MS/MS) analysis was performed on a Sciex Applied Biosystems Qtrap 4000 triple-quadrupole mass spectrometer coupled to an Agilent 1260 HPLC system to quantify the biological samples. Chromatography for RIF, INH, and Acetyl-INH was performed on an Thermo Hypersil Betasil C8 (2.1x50 mm; particle size, 5 μm) using a reverse phase gradient elution. The gradient used 0.1% formic acid and 0.01% Heptafluorobutyric Acid (HFBA) in Milli-Q deionized water for the aqueous mobile phase and 0.1% formic acid 0.01% HFBA in acetonitrile for the organic mobile phase. RIF-d8, INH-d4, and Acetyl-INH-d4 were used as internal standards (IS). The compounds were ionized using ESI positive mode ionization and monitored using masses RIF (823.50/791.60), RIF-d8 (831.50/799.60), INH (252.20/80.30), INH-d4 (256.20/84.30), Ac-INH (180.40/121.00), and Ac-INH-d4 (184.40/142.10).

### Statistics

Two-tailed Unpaired Student *t* test with Welch-correction, 1-way and 2-way ANOVA with Šidák multiple comparison tests were conducted in Prism (GraphPad). All experiments were repeated at least twice. Number of mice used in each experiment is indicated in Figure legends.

## Supporting information

S1 Fig H&E stain of TDM granulomas and TB granulomas with and without Marimastat. Scale bar: 100μm.
(TIFF)

S2 Fig CFU count of lung tissue from Mtb-infected mice treated with PBS or Marimastat after 6-week’s infection (n = 5). Data represented mean. *: p < 0.05, One-way ANOVA with Šidák multiple comparison test.
(TIFF)

S3 Fig CFU count of BMDMs infected with Mtb and treated with PBS or Marimastat for 2 or 4 days (n = 5). Data represented mean ± SD. Two-way ANOVA with Šidák multiple comparison test.
(TIFF)

S4 Fig Collagen content in TDM matrigel (A) and TB matrigel (B) with Marimastat and MMP-2 inhibitor II treatment (n = 5) from infected mice under different treatments (n = 5). Data represented mean ± SD. *: p < 0.05, **: p < 0.01, ***: p < 0.001, One-way ANOVA with Šidák multiple comparison test.
(TIFF)

S5 Fig (A): Protein level of MBL in other sets of Mtb infected mice under different treatments (n = 5). (B): MMP-9 and MMP-2 protein level from lung lyse of infected mice with different treatments (n = 5) in two separate experiments.
(TIFF)

S6 Fig Number of α-SMA positive blood vessels at 2-week and 4-week post infection from animal treated with PBS or Marimastat (n = 5). Data represented mean ± SD. *: p < 0.05, One-way ANOVA with Šidák multiple comparison test.
(TIFF)

S7 Fig RIF and INH were injected intravenously to infected animals treated with or without Marimastat treatment. Animals were sacrificed 1h, 2h and 4h post injection. RIF and INH concentrations were measured in the lung (A, C) and the plasma (B, D). Data represented mean ± SD. **: p < 0.01, ****: p < 0.0001, One-way ANOVA with Šidák multiple comparison test.
(TIFF)
