# Supplementary figures and images for: Matrix metalloproteinase inhibitors enhance the efficacy of frontline drugs against Mycobacterium tuberculosis
Source: PLoS Pathog. 2018 Apr 26;14(4):e1006974. doi: 10.1371/journal.ppat.1006974 (PMC5919409; doi:10.1371/journal.ppat.1006974)

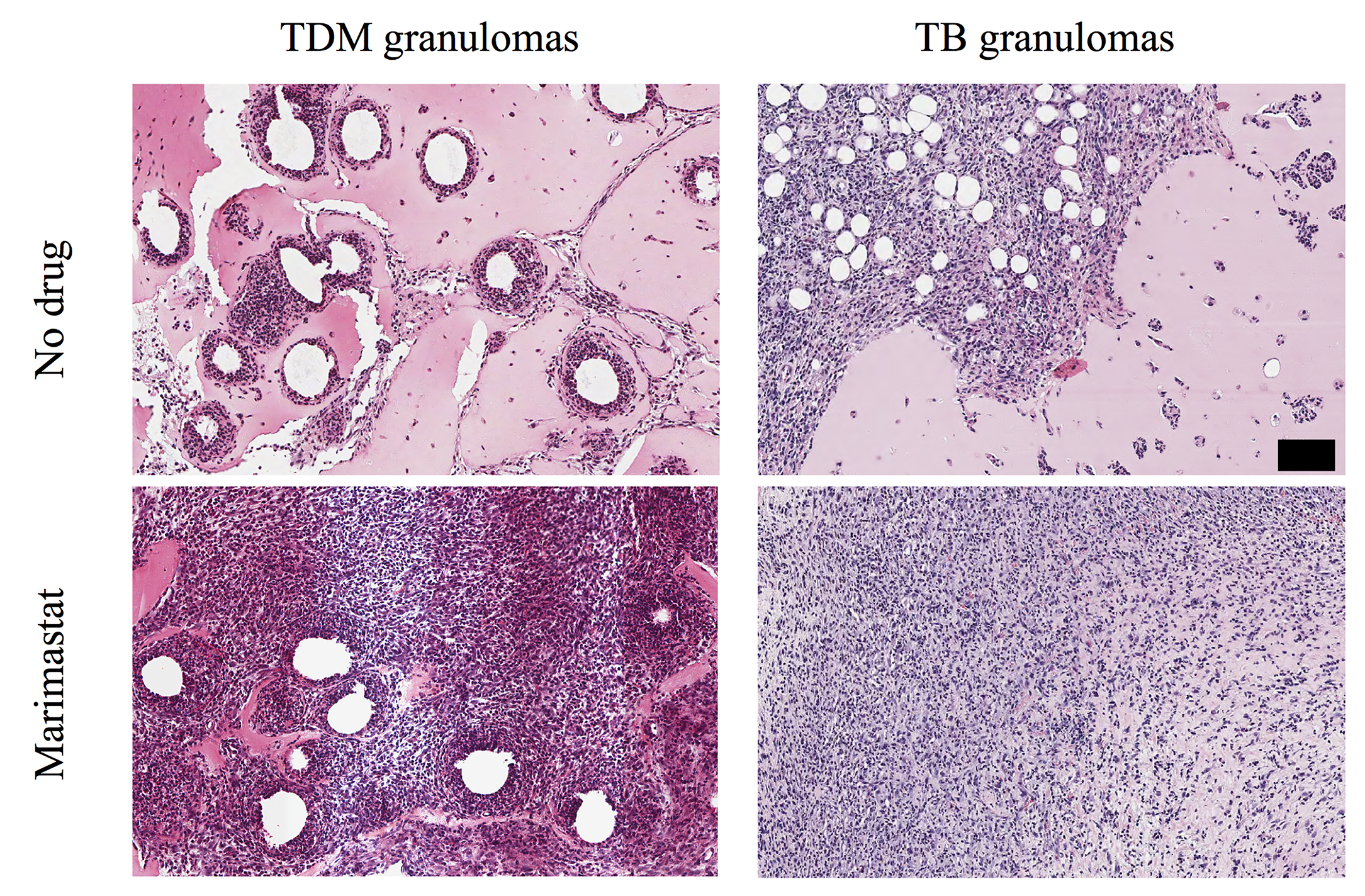

Supplement: S1 Fig — Scale bar: 100μm. (TIFF) [file ppat.1006974.s001.tiff]

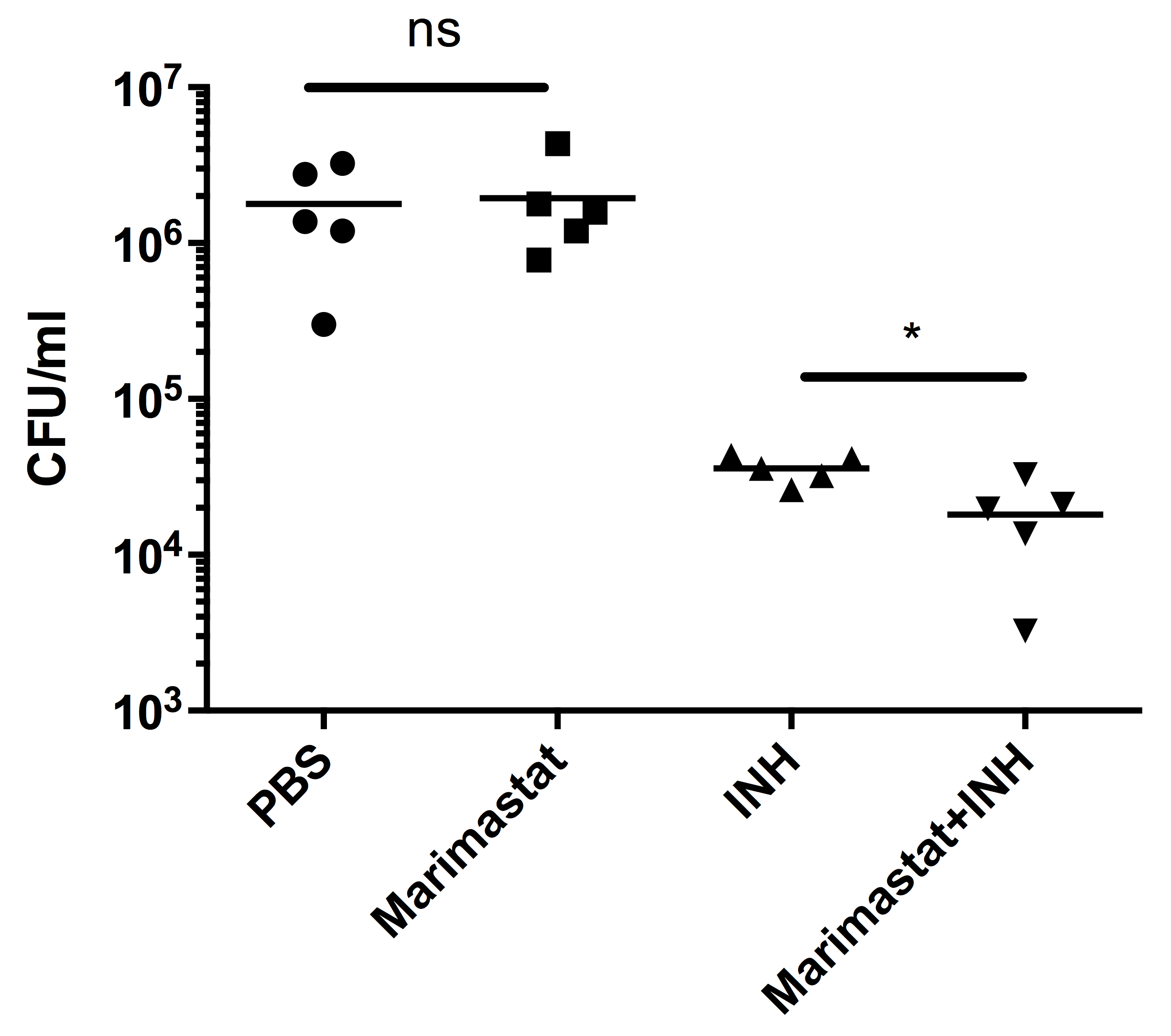

Supplement: S2 Fig — Data represented mean. *: p < 0.05, One-way ANOVA with Šidák multiple comparison test. (TIFF) [file ppat.1006974.s002.tiff]

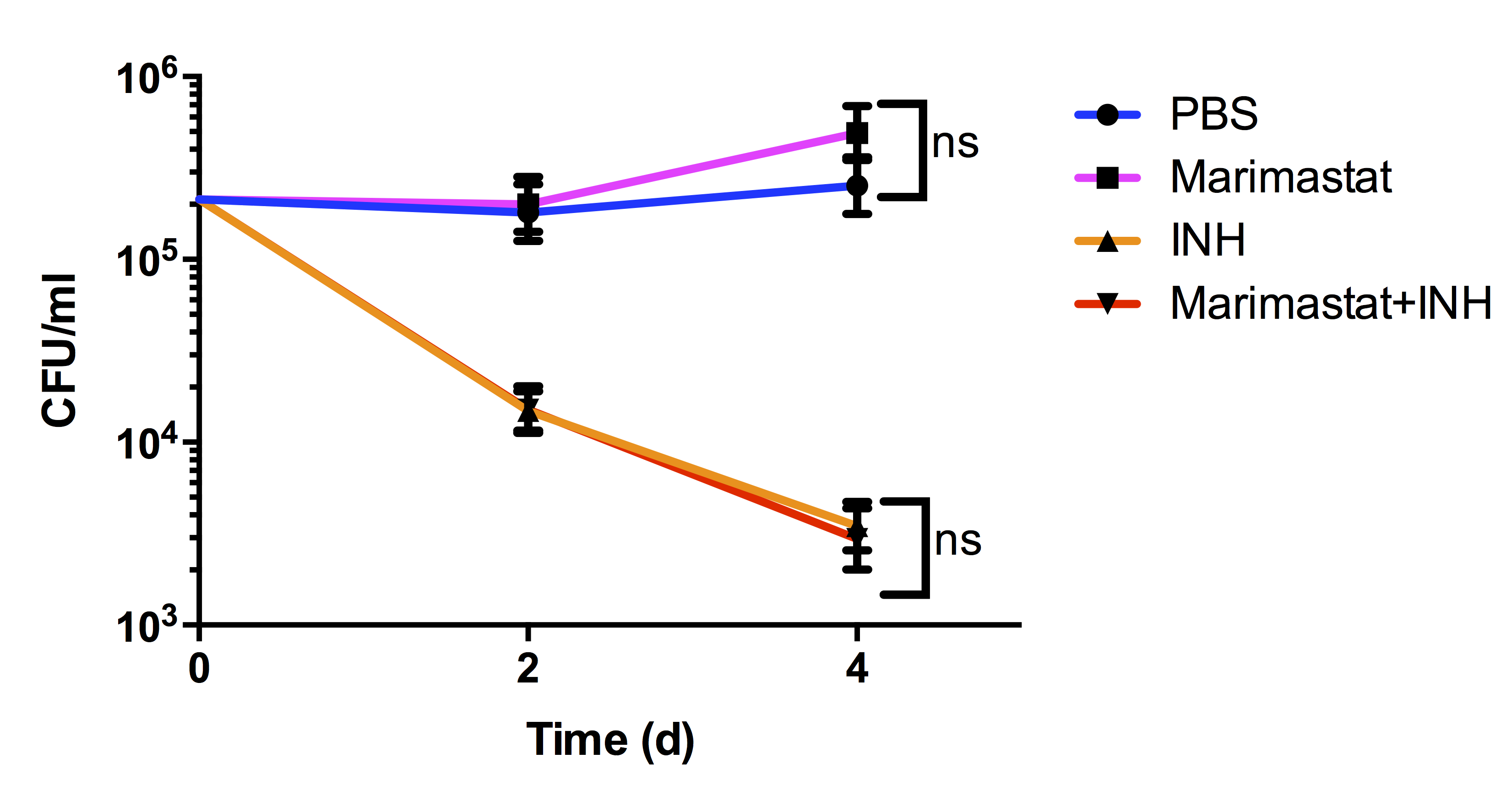

Supplement: S3 Fig — Data represented mean ± SD. Two-way ANOVA with Šidák multiple comparison test. (TIFF) [file ppat.1006974.s003.tiff]

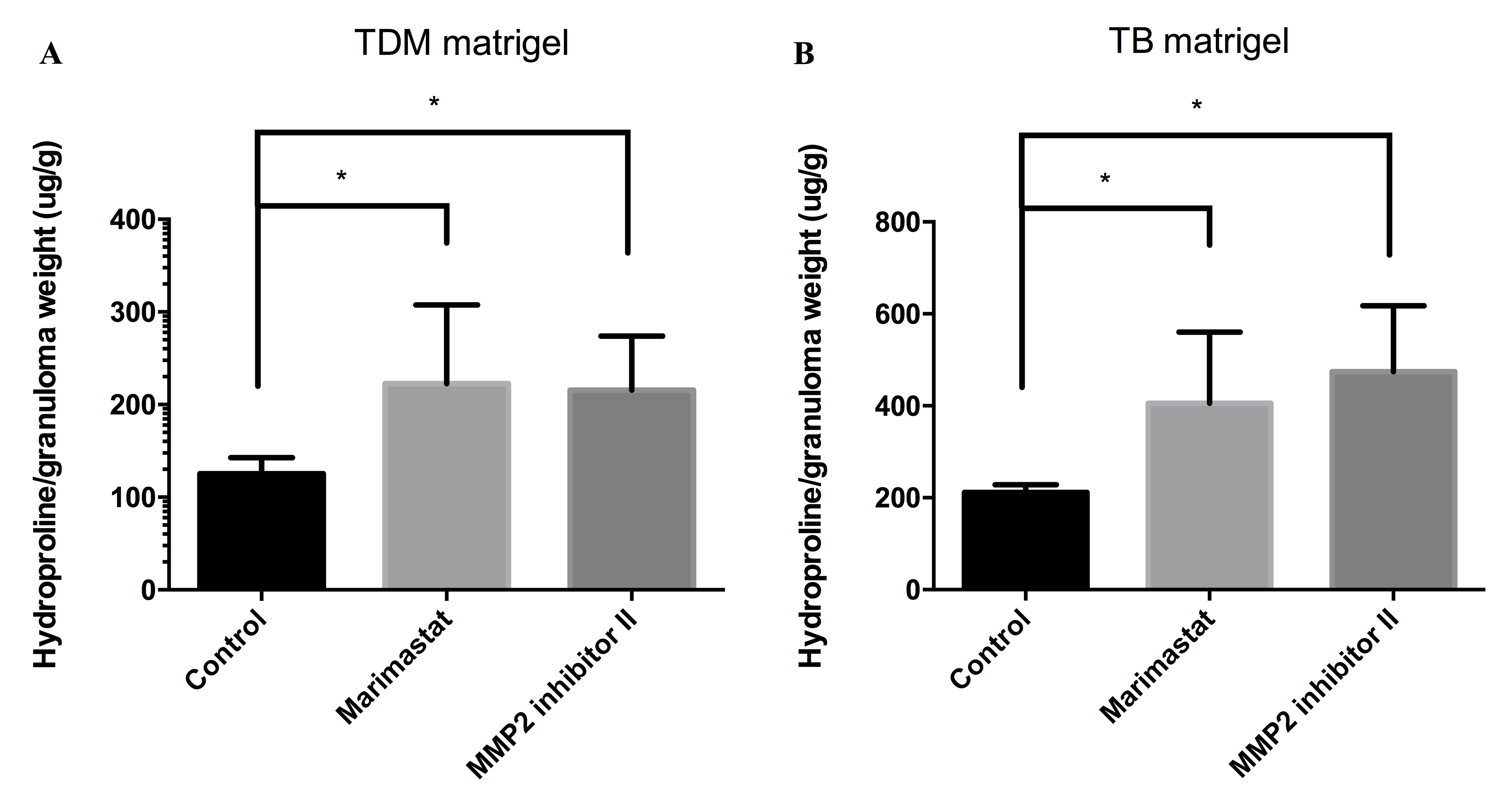

Supplement: S4 Fig — Collagen content in TDM matrigel (A) and TB matrigel (B) with Marimastat and MMP-2 inhibitor II treatment (n = 5) from infected mice under different treatments (n = 5). Data represented mean ± SD. *: p < 0.05, **: p < 0.01, ***: p < 0.001, One-way ANOVA with Šidák multiple comparison test. (TIFF) [file ppat.1006974.s004.tiff]

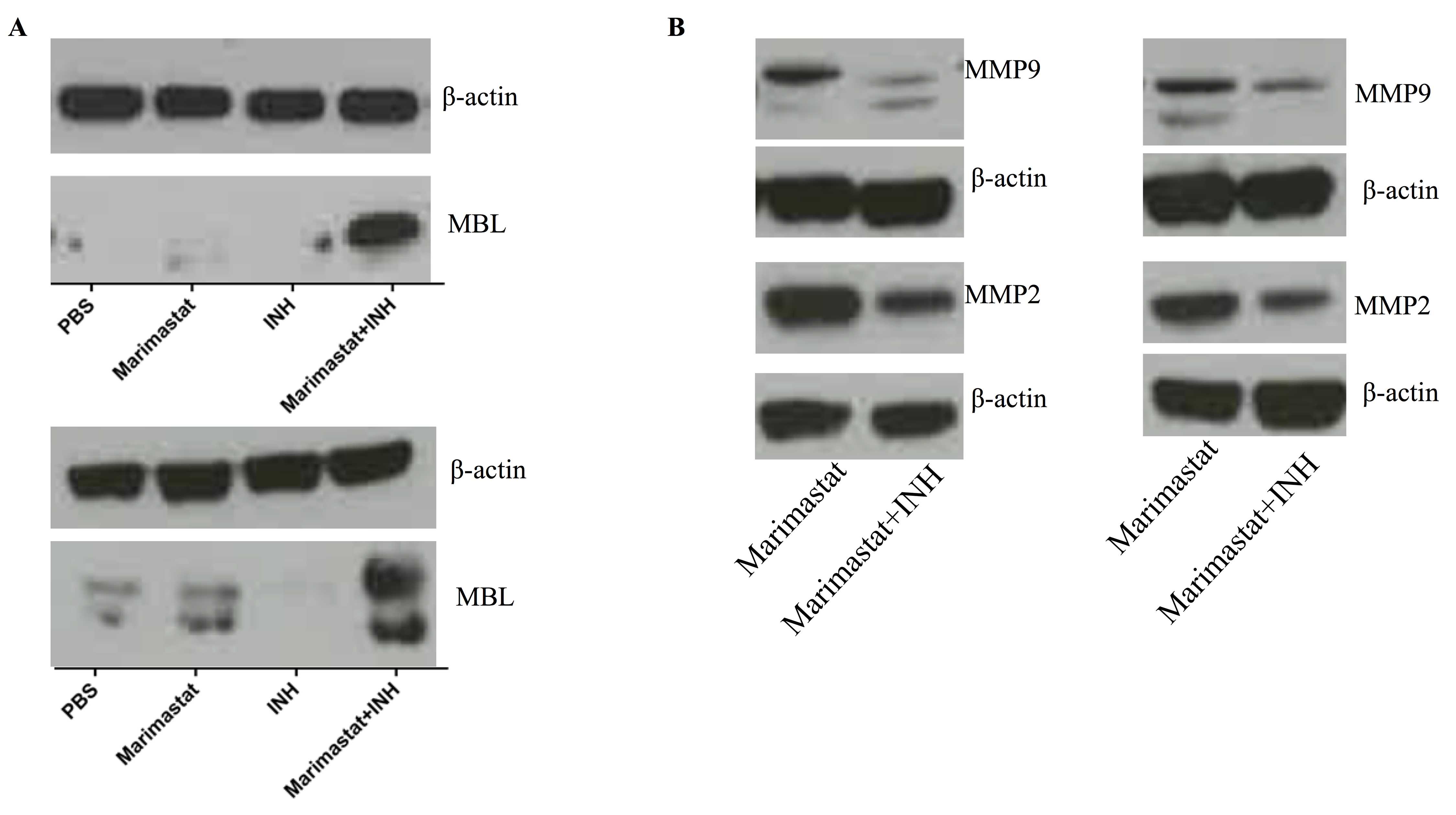

Supplement: S5 Fig — (A): Protein level of MBL in other sets of Mtb infected mice under different treatments (n = 5). (B): MMP-9 and MMP-2 protein level from lung lyse of infected mice with different treatments (n = 5) in two separate experiments. (TIFF) [file ppat.1006974.s005.tiff]

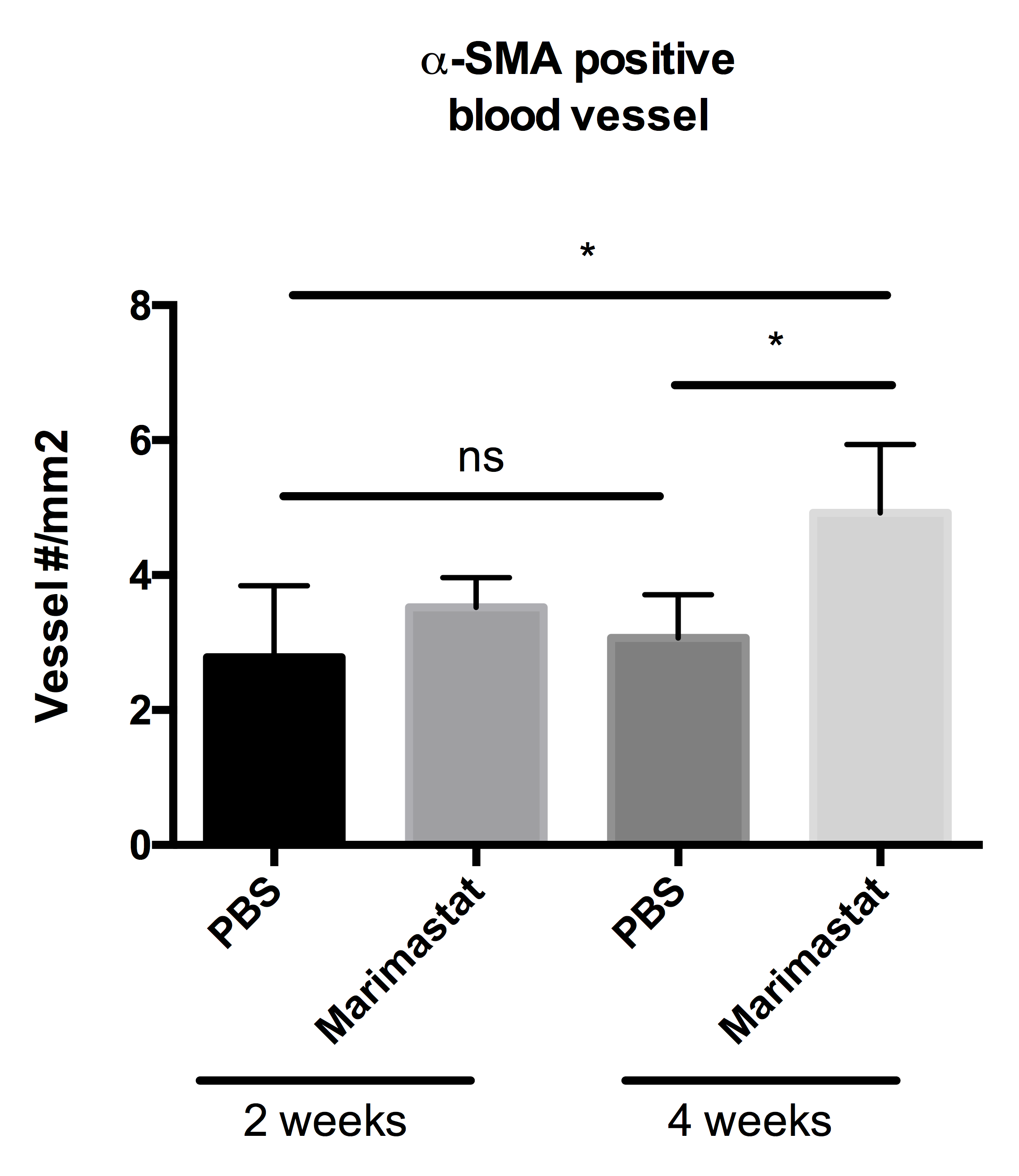

Supplement: S6 Fig — Data represented mean ± SD. *: p < 0.05, One-way ANOVA with Šidák multiple comparison test. (TIFF) [file ppat.1006974.s006.tiff]

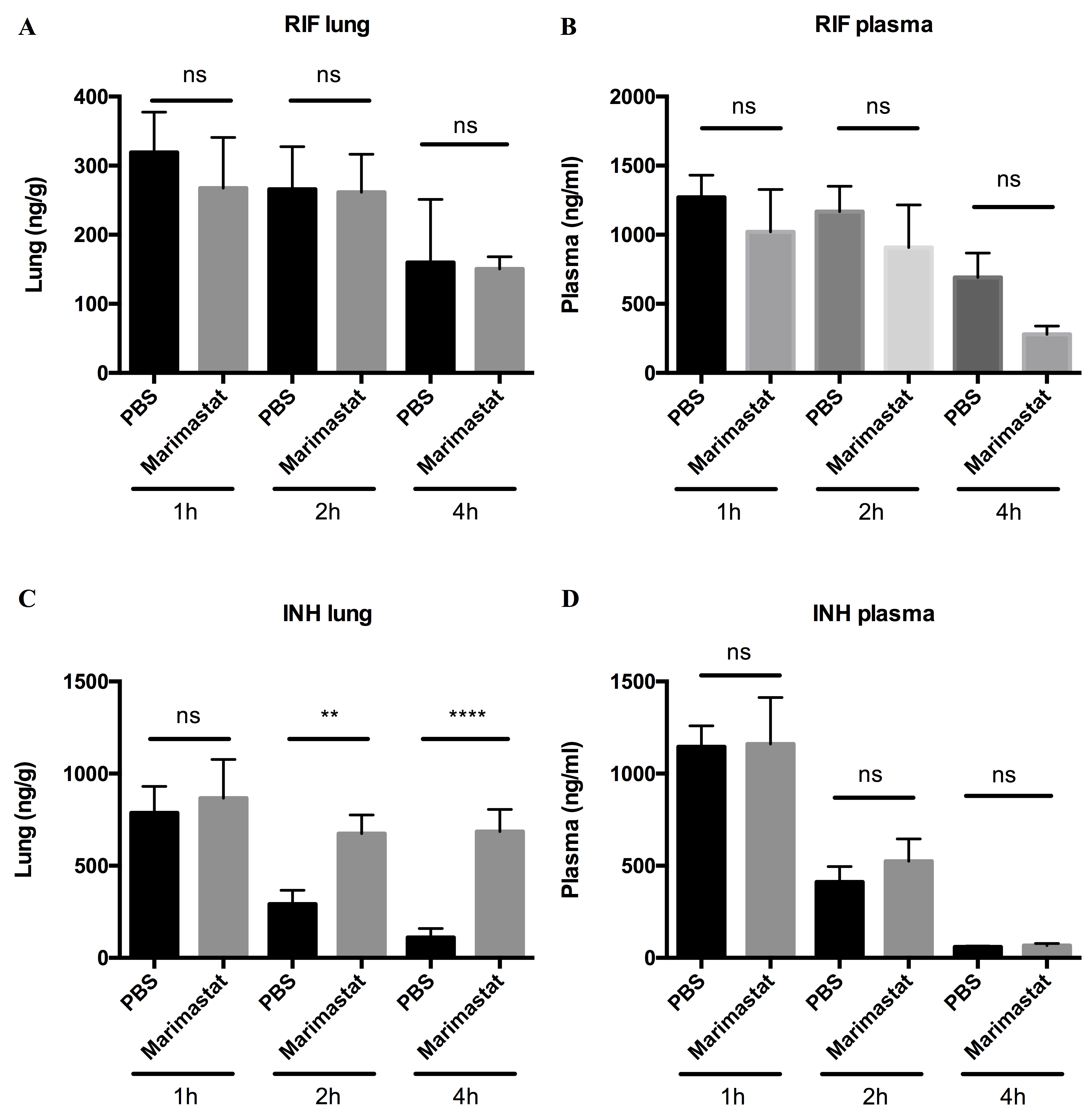

Supplement: S7 Fig — Animals were sacrificed 1h, 2h and 4h post injection. RIF and INH concentrations were measured in the lung (A, C) and the plasma (B, D). Data represented mean ± SD. **: p < 0.01, ****: p < 0.0001, One-way ANOVA with Šidák multiple comparison test. (TIFF) [file ppat.1006974.s007.tiff]
